# Strain Analysis for Early Detection of Fibrosis in Arrhythmogenic Cardiomyopathy: Insights from a Preliminary Study

**DOI:** 10.3390/jcm13237436

**Published:** 2024-12-06

**Authors:** Valeria Pergola, Marika Martini, Filippo Amato, Dan Alexandru Cozac, Petra Deola, Ilaria Rigato, Giulia Mattesi, Maria Teresa Savo, Eleonora Lassandro, Vittorio Marzari, Simone Corradin, Giorgio De Conti, Martina Perazzolo Marra, Raffaella Motta, Barbara Bauce

**Affiliations:** 1 Department of Cardiac, Thoracic, Vascular Sciences and Public Health, University of Padova, 35128 Padova, Italy; valeria.pergola@gmail.com (V.P.); eleonora.lassandro@studenti.unipd.it (E.L.); vittorio.marzari@studenti.unipd.it (V.M.); barbara.bauce@unipd.it (B.B.); 2 University of Medicine, Pharmacy, Science and Technology “George Emil Palade”, Gheorghe Marinescu 38, 540142 Târgu Mureș, Romania; 3 Fondazione Poliambulanza Istituto Ospedaliero Area Cardiovascolare, Via Leonida Bissolati, 57, 25124 Brescia, Italy

**Keywords:** arrhythmogenic cardiomyopathy (ACM), global longitudinal strain (GLS), late enhancement (LE)

## Abstract

**Background:** Arrhythmogenic cardiomyopathy (ACM) is a genetic disorder characterized by fibrofatty replacement of myocardial tissue, predominantly affecting the right ventricle (RV), but often involving the left ventricle (LV) as well. The early detection of fibrosis, crucial for risk stratification, has been enhanced by advanced imaging techniques. Global longitudinal strain (GLS) has shown promise as a surrogate marker for late enhancement (LE) in identifying myocardial fibrosis, yet precise cut-off values for strain are lacking. The aim of the study is to evaluate LV strain as a predictor of LE in ACM and to define strain cut-offs for early fibrosis detection, enhancing non-invasive diagnostic accuracy. **Methods:** This retrospective single-center study included 64 patients diagnosed with ACM. Echocardiographic analysis using speckle-tracking echocardiography was performed to assess LV strain. LE was evaluated through cardiac magnetic resonance (CMR) or via cardiac computed tomography (CCT) in cases with CMR contraindications. The study aimed to correlate regional LV strain values with the presence of LE, identifying cut-off values predictive of fibrosis. **Results:** The study found significant correlations between reduced LV strain values and the presence of LE, particularly in the anterolateral and inferolateral segments (*p* < 0.05). Specific strain thresholds, such as those for segment 12 (*p* = 0.02) and segment 17 (*p* = 0.03), were identified as predictive markers for LE. These findings suggest that strain imaging could serve as a non-invasive tool for the early detection of myocardial fibrosis in ACM patients. **Conclusions:** LV strain analysis offers potential as a non-invasive surrogate marker for myocardial fibrosis in ACM. Incorporating strain imaging into routine echocardiographic evaluations could improve early diagnosis and risk stratification, guiding patient management.

## 1. Introduction

Arrhythmogenic cardiomyopathy (ACM) is a genetic disorder characterized by the replacement of myocardial tissue with fibrofatty deposits, predominantly affecting the right ventricle (RV), but often involving the left ventricle (LV) as well [1]. This condition is associated with an increased risk of life-threatening arrhythmias and sudden cardiac death (SCD), making early diagnosis and risk stratification crucial [2]. Diagnostic criteria initially considered mainly the traditional right-sided form of the disease (Arrhythmogenic right ventricular cardiomyopathy: ARVC) focusing on the imaging detection of RV dilatation and kinetic alterations as well as on histological findings [3]. However, with the growing recognition of LV involvement in the disease and the emerging role of cardiac magnetic resonance (CMR) in tissue characterization, the formulation of the new Padua criteria has become necessary [4]. Additionally, current advancements in echocardiography have opened new avenues for detecting subclinical myocardial dysfunction, offering further promising diagnostic potential. In particular, global longitudinal strain (GLS) and regional strain measurements, which provide quantitative assessments of myocardial deformation, have demonstrated increased sensitivity in detecting patients with ACM, both in its RV and LV forms, in comparison with traditional echocardiographic parameters [5,6]. Recent evidence suggests that GLS could serve as a potential proxy for late enhancement (LE) in detecting ACM [7], with important diagnostic and prognostic implications. The major advantage of GLS is that it offers a more convenient and accessible method compared to CMR imaging; however, precise cut-off values, which are necessary for more reliable application in clinical practice, have not yet been established. Furthermore, for patients who cannot undergo CMR due to contraindications, LE can also be detected using cardiac computed tomography (CCT) as late iodine enhancement (LIE), providing an alternative imaging modality [8].

The primary aim of this study is to assess the utility of LV strain measurements in predicting LE in ACM patients. The study seeks to establish cut-off values for regional LV strain that can reliably predict LE, facilitating early detection and better risk stratification of myocardial fibrosis in ACM. By correlating strain values with LE, the study aims to develop a non-invasive diagnostic tool to enhance the sensitivity and specificity of current ACM diagnostic criteria, ultimately improving patient management and outcomes.

## 2. Materials and Methods

### 2.1. Study Population

This was a retrospective, single-center study conducted at the Cardiomyopathy Unit of Padua University Hospital. The study included 64 patients who had a confirmed diagnosis of ACM according to both the 2010 Revised Task Force Criteria [3] and the Padua criteria [4]. Exclusion criteria were applied to ensure the quality and reliability of the echocardiographic analysis and included the presence of atrial fibrillation and suboptimal echocardiographic image quality for speckle-tracking echocardiography (STE) analysis. Additionally, patients with contraindications to CMR imaging underwent CCT to detect LE. Complete data sets, including both strain measurements and CMR/CT scans, were available for 57 patients. Specifically, CMR data were available for 52 patients, while CCT scans were available for 5 patients. The study protocol was approved by the local Ethical Committee (approval number: 507n/AO/24, date 4 July 2024).

Demographic and clinical data were obtained from hospital records at the time of the echocardiographic examination. LE via CMR or CCT scan was included in the analysis if the imaging had been performed within six months prior to the reference echocardiogram. This time frame was chosen to maintain consistency in the correlation between echocardiographic and imaging findings, although a discussion of potential variability in disease progression during this period could provide additional context.

### 2.2. Echocardiographic Evaluation

All patients underwent transthoracic echocardiography following a standardized protocol [9]. All patients included in the analysis were in sinus rhythm at the time of echocardiographic evaluation, to ensure accurate and consistent measurements. Resting two-dimensional echocardiography was performed in accordance with the guidelines of the European Association of Echocardiography and the American Society of Echocardiography [9,10], using either a Vivid E80 machine (GE Healthcare, Chicago, IL, USA) or a Philips EPIC 7 machine (Philips Healthcare, Amsterdam, The Netherlands). These recordings were digitally stored for offline analysis on a specialized workstation. An independent and experienced doctor, blinded to the patients’ medical histories, analyzed all of the echocardiograms. This blinding was crucial to avoid bias in the interpretation of the results. RV measurements were performed following current recommendations [11]. LV GLS was assessed in three apical views using two-dimensional speckle-tracking echocardiography (STE) [12]. The frame rate for the recordings ranged from 45 to 75 frames per second, ensuring adequate temporal resolution for strain analysis. Strain analysis was conducted offline using the TomTec Image Arena (TTA2 Build 468168, Cardiac Performance Analysis software, V1.4.0.109, Philips Medical Systems, The Netherlands). GLS values were recorded using a 17-segment model and determined as the average of these segments. Manual adjustments to the region of interest were made to ensure accurate coverage of the myocardial wall.

RV global longitudinal strain analysis (RVGLS) was measured in the RV-focused apical four-chamber view according to current guidelines [13]. LV mechanical dispersion (MD) was defined as the standard deviation of time from Q/R on surface ECG to peak negative strain in the 17 LV segments [14].

### 2.3. Cardiac Magnetic Resonance Imaging

CMR images were acquired using a 1.5 Tesla scanner (Magnetom Avanto, Siemens Healthineers, Erlangen, Germany) equipped with ECG triggering and a phased array coil system. The detailed study protocol, imaging techniques, and post-processing analysis have been described in a previous publication by our group [15]. For this study, the presence and pattern of LGE were retrospectively evaluated by cardiologists and radiologists with expertise in CMR. Importantly, these evaluations were performed by clinicians blinded to both the clinical and echocardiographic data, ensuring unbiased interpretation. The scans included in the study were conducted within six months of the echocardiographic assessment, although potential temporal discrepancies in disease manifestation are not explicitly addressed.

### 2.4. Cardiac Computed Tomography Imaging

For patients unable to undergo CMR due to contraindications, late enhancement imaging was performed using a multi-detector CT scanner. Specifically, CCT imaging was conducted with a 320-slice MDCT scanner (Aquilion One; Canon Medical Systems, Otawara, Japan). Our CCT protocol has been previously described [16]. CCT LIE images were reviewed by two experienced physicians using a narrow window setting to enhance contrast. Adjustments to the window settings were made according to the tube voltage and virtual monochromatic imaging (VMI) energy levels [17], optimizing image quality. Notably, the left ventricular inferior wall, which is prone to streak artifacts from the diaphragm, was carefully evaluated. In cases of discordant findings, a third experienced physician was consulted to ensure accurate interpretation.

### 2.5. Statistical Analysis

Continuous variables were presented as mean ± standard deviation (SD) when normally distributed, or as median and interquartile range (IQR) when the distribution was non-normal. Categorical variables were expressed as absolute values and percentages (n, %). To evaluate the diagnostic performance of regional strain measurements in predicting LE, the sensitivity, specificity, positive predictive value (PPV), and negative predictive value (NPV) were calculated for each regional strain cut-off value.

The Youden index, which optimizes the balance between sensitivity and specificity, was employed to determine the optimal strain thresholds for predicting the presence of LGE. This analysis was performed for each of the 17 myocardial segments, allowing for a comprehensive assessment of regional myocardial function. The use of the Youden index helps in identifying the most appropriate cut-off points, which are critical for the potential clinical application of strain measurements in diagnosing ACM.

To assess the association between regional myocardial strain and the presence of LGE, univariate logistic regression analysis was performed for each myocardial segment.

All statistical analyses were conducted using GraphPad Prism software (version 8.0.2, GraphPad Software, San Diego, CA, USA). All statistical tests were two-sided, and a *p*-value of less than 0.05 was considered statistically significant, indicating a threshold for rejecting the null hypothesis.

## 3. Results

### 3.1. Baseline Clinical Characteristics

The study included a total of 64 patients diagnosed with ACM. The mean age of the participants was 42 ± 15 years, with a predominance of male patients (71.8%). The majority of the cohort exhibited a biventricular form of ACM (53.1%), followed by left-dominant (26.5%) and right-dominant forms (20.3%) as classified by the Padua criteria [4]. A significant proportion of the patients (54.6%) had a family history of ACM, and 17.1% had a family history of SCD. Detailed baseline clinical characteristics are presented in Table 1.

### 3.2. Echocardiographic Parameters

Echocardiographic assessment revealed a mean LVEF of 58% (range 52–62), indicating preserved systolic function in the study population. However, the GLS of the left ventricle was slightly reduced, with a mean value of −17.9 ± 3.3%. The RVGLS was further reduced to −15.2% (range −12.0 to −19.6%). The average mechanical dispersion (MD), indicative of heterogeneity in myocardial contraction, was 56 ms (IQR 44–67.7). These echocardiographic parameters are summarized in Table 2.

### 3.3. Regional Myocardial Strain

Regional strain analysis of the LV revealed lower strain values, particularly in the inferior and inferolateral walls, as shown in Table 3.

### 3.4. Late Enhancement and Myocardial Strain Evaluation

LE, a marker of myocardial fibrosis, was observed in various myocardial segments, with the highest prevalence in the inferolateral and anterolateral segments (Table 4).

Cut-off values for regional left ventricular strain that predict the presence of LE were identified using Receiver Operating Characteristic (ROC) curves and the Youden index. These cut-off values are detailed in Table 5, with segment-specific thresholds for predicting LGE.

Univariate logistic regression analysis confirmed that several left ventricular segments showed significant associations between reduced strain values and the presence of LE. Notably, segments 12 (>−14.5%), 16 (>−14.2%), and 17 (>−18.5%) demonstrated significant predictive value for LGE (*p* < 0.05), as shown in Table 6. These findings underscore the potential utility of regional strain analysis in identifying myocardial fibrosis in ACM, particularly in segments prone to fibrofatty infiltration.

**Figure 1:**
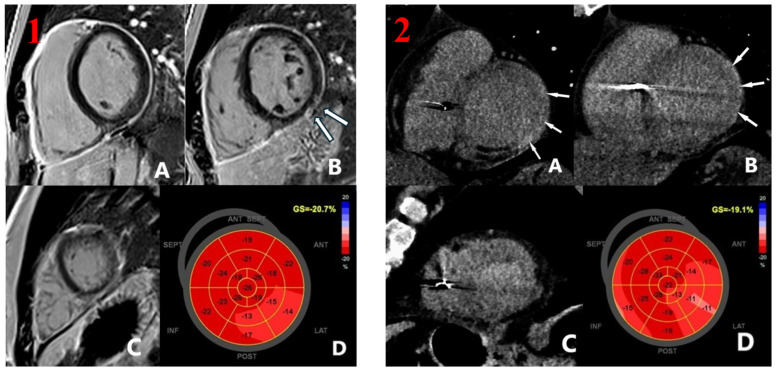
**(1) Cardiac Magnetic Resonance**: short-axis PSIR (Phase-Sensitive Inversion Recovery) sequences, depicting basal (**A**), mid-ventricular (**B**), and apical levels (**C**), with late gadolinium enhancement observed in the medium inferolateral and inferior walls (**B**), corresponding to a reduction in regional strain (**D**). **(2) Cardiac Computed tomography**: post-contrast sequences showing basal (**A**), mid-ventricular (**B**), and apical levels (**C**), with late iodine enhancement observed in the basal and medium lateral and inferolateral walls (**B**), corresponding to a reduction in regional strain (**D**).

Figure 1 shows an example of correlation between regional strain and LGE/LIE.

## 4. Discussion

In our study, we identified significant regional impairments in LV strain, particularly in the anterolateral and inferolateral walls, and established specific GLS cut-off values predictive of LE. These findings provide a non-invasive method for detecting myocardial fibrosis, aligning with prior evidence that LV strain abnormalities precede overt clinical dysfunction. By demonstrating a strong correlation between reduced GLS and the presence of LE, our study highlights the diagnostic and prognostic utility of LV strain analysis, which complements and extends the current understanding of LV involvement in ACM. In ACM, LV involvement, whether in isolated left-dominant or biventricular forms, is primarily detected by the presence of fibrosis in the context of preserved LVEF, since fibro-fatty replacement of the myocardium often precedes wall motion impairment [18]. STE has gained recognition for its sensitivity in detecting early impairments in LV longitudinal contractility, even when the ejection fraction remains within normal limits, as demonstrated in various cardiac conditions [19,20]. Although much of the existing literature has focused on RV strain [21,22], the role of LV strain in ACM has been less explored. Namasivayam et al. showed that the assessment of RV strain enhanced the diagnostic accuracy beyond the current echocardiographic criteria by detecting patients who would otherwise be missed but still meet the diagnostic criteria for ARVC. In contrast, while LV strain did not significantly enhance diagnostic accuracy, it did demonstrate prognostic value [5]. While our study focused on LV strain analysis and its correlation with late enhancement (LE), we acknowledge the potential value of integrating RV strain into a comprehensive diagnostic framework for ACM. Previous studies have demonstrated the diagnostic and prognostic utility of RV strain, particularly in identifying patients who might otherwise be missed by traditional criteria [5,6]. In our cohort, RV strain values were reduced and consistent with findings from the existing literature. However, the evaluation of LGE in the thin RV wall remains challenging due to its limited thickness and the potential overlap with fatty tissue [23]. This limitation may explain why LGE is not yet included in the Task Force Criteria. For this reason, we prioritized the more robust assessment of LV fibrosis and focused solely on deriving LV GLS cut-off values. Our study contributes several novel insights. Firstly, we performed an in-depth analysis of regional wall strain across individual myocardial segments, revealing significantly impaired strain values in the anterolateral and inferolateral walls. These findings are consistent with the existing literature, which identifies these regions as particularly vulnerable to fibrotic changes [6]. The early detection of myocardial involvement is crucial in ACM, a condition where sudden cardiac death can occur even in the absence of fully satisfied diagnostic criteria [24].

Moreover, we established a strong correlation between reduced GLS values and the presence of LE, identifying specific GLS regional cut-off values that are predictive of fibrosis. This provides a non-invasive method for detecting myocardial fibrosis, which is particularly beneficial for patients during follow-up. While GLS analysis via STE is a practical and widely accessible tool for detecting myocardial strain abnormalities, it has certain limitations compared to feature tracking CMR. GLS measurements are more susceptible to variations in image quality, operator dependency, and the limitations of 2D imaging [25]. In contrast, feature tracking CMR offers higher spatial resolution and superior accuracy, particularly for assessing myocardial strain in thinner ventricular walls [26]. However, the ease of access, lower cost, and widespread availability of STE make it a valuable alternative for routine clinical use, particularly in settings where advanced imaging modalities like CMR are not feasible.

While therapeutic measures were not the focus of this study, we acknowledge the potential role of mineralocorticoid receptor antagonists (MRAs) in mitigating cardiac fibrosis and arrhythmogenicity in ACM. Recent studies have highlighted their antifibrotic effects and their potential to improve arrhythmic outcomes in this patient population [27]. Future research exploring the interplay between strain analysis and therapeutic interventions, such as MRAs, could provide valuable insights and further refine risk stratification and management strategies for ACM patients.

Our findings align with previous research by Shen et al. [28], which demonstrated that LV deformation analysis via feature tracking was more sensitive than LVEF in detecting early LV dysfunction in ACM and provided significant prognostic value. A recent echocardiographic study further demonstrated the potential of LV strain impairment as a surrogate marker for LGE presence, particularly in patients who exhibit fibrosis without overt clinical manifestations [7]. However, unlike our study, that research did not establish specific cut-off values, which are critical for improving the diagnostic precision of strain imaging.

The observed correlation between LV strain abnormalities and LE in our study underscores the potential of strain imaging as a valuable non-invasive marker for detecting myocardial fibrosis. Consequently, we advocate for the routine integration of LV strain assessment into echocardiographic evaluations for patients with confirmed or suspected ACM. This approach could be particularly useful in the early diagnosis of the disease and in guiding the referral of patients for advanced imaging when clinical suspicion arises.

However, our study has certain limitations. The retrospective design and relatively small sample size may limit the generalizability and robustness of the identified cut-off values and logistic regression models. Moreover, we acknowledge the six-month interval between imaging and echocardiographic evaluations, but this consistent time frame across patients likely minimized the impact of potential disease progression. Additionally, our analysis focused solely on GLS, which reflects the practical application of GLS in clinical settings. In line with the purpose of our study, we did not focus on RV strain and mechanical dispersion; however, these parameters exhibited reduced values consistent with the existing literature [21,29]. This study did not directly assess the prognostic implications of strain cut-offs, though the correlation with fibrosis, a known marker of adverse outcomes, supports their clinical relevance. This is a preliminary study; multicenter studies are needed to validate our findings and to further explore the prognostic implications of LV strain in ACM.

## 5. Conclusions

In conclusion, our study highlights the potential of LV strain analysis as a non-invasive marker for detecting myocardial fibrosis in patients with ACM. The significant correlation between strain abnormalities and LE supports the inclusion of strain imaging in routine echocardiographic evaluations, particularly for early diagnosis and risk stratification in patients with suspected ACM. By integrating strain analysis into clinical practice, clinicians may improve the early identification of myocardial involvement, thereby enhancing patient management and outcomes in this high-risk population.

## Figures and Tables

**Table 1:** Baseline clinical characteristics.

Parameters	Overall (n = 64 Patients)
Age (years)	42 ± 15
Male gender (n, %)	46 (71.8%)
Body surface area (BSA), (kg/m^2^)	1.8 ± 0.1
**Types of arrhythmogenic cardiomyopathy**	
Right dominant (n, %)	13 (20.3%)
Biventricular (n, %)	34 (53.1%)
Left dominant (n, %)	17 (26.5%)
**Cardiovascular risk factors**	
Family history of arrhythmogenic cardiomyopathy (n, %)	35 (54.6%)
Family history of sudden cardiac death (n, %)	11 (17.1%)
Arterial hypertension (n, %)	5 (7.8%)
Dyslipidemia (n, %)	9 (14.0%)
Smoking (n, %)	1 (1.5%)
Diabetes mellitus (n, %)	1 (1.5%)
Inflammatory/autoimmune disease (n, %)	3 (4.6%)
**Clinical presentation**	
Chest pain (n, %)	1 (1.5%)
Palpitations (n, %)	4 (6.2%)
Dyspnea (n, %)	1 (1.5%)
Supraventricular tachycardia (n, %)	2 (3.1%)
Ventricular tachycardia (n, %)	17 (26.5%)
Ventricular fibrillation (n, %)	6 (9.3%)
Syncope (n, %)	12 (18.7%)
Implantable cardioverter defibrillator (n, %)	28 (43.7%)
Primary prevention of sudden cardiac death (n, %)	14 (21.8%)
Coronary artery disease (n, %)	0 (0%)
Previous percutaneous coronary intervention (n, %)	0 (0%)
Beta blockers (n, %)	54 (84.3%
Calcium antagonists (n, %)	2 (3.1%)
Renin–angiotensin–aldosterone inhibitors (n, %)	13 (20.3%)
Antialdosteronic (n, %)	2 (3.1%)
Angiotensin receptor/neprilysin inhibitor (n, %)	1 (1.5%)
Amiodarone (n, %)	4 (6.2%)

Values are expressed as mean ± SD or median and IQR as needed, or n (%).

**Table 2:** Echocardiographic characteristics.

Echocardiographic Characteristics	Overall (n = 64)
Interventricular septum (mm)	9 (8–10)
Telediastolic left ventricle diameter (mm)	51 (45–55)
Posterior wall (mm)	8 (7–9)
Left ventricle telediastolic volume indexed (mL/m^2^)	66 ± 15
Left ventricle ejection fraction (%)	58 (52–62)
Indexed right ventricle area	15.2 (12.0–19.6)
Fractional area change (%)	35 ± 10
Tricuspid annular plane systolic excursion (mm)	21 (18–24)
S’ Right ventricle (cm/s)	10 (8.8–12.0)
Left ventricle global longitudinal strain (%)	−17.9 ± 3.3
Mechanical dispersion (ms)	56 (44–67.7)
Myocardial work index (mmHg%)	1609 (1074–1906)
Constructive myocardial work (mmHg%)	1711 (1318–2055))
Myocardial work wasted (mmHg%)	96 (73.8–174.5)
Myocardial work efficiency (%)	94 (89.5–95.5)
Global right ventricle strain	−16.7 (−19.4–−11.4)

Values are expressed as mean ± SD or median and IQR as needed, or n (%).

**Table 3:** Regional left ventricular myocardial strain (mean ± standard deviation).

	Basal	Mid	Apical
Anterior	−16.6 ± 4.0	−19.2 ± 4.7	−18.0 ± 5.7
Anteroseptal	−17.1 ± 4.4	−19.6 ± 5.0	−22.4 ± 6.5
Inferoseptal	−19.4 ± 4.7	−18.7 ± 5.2	
Inferior	−17.2 ± 5.9	−16.3 ± 4.0	−20.7 ± 6.6
Inferolateral	−16.0 ± 6.2	−16.8 ±4.9	−18.5 ± 5.6
Anterolateral	−18.3 ± 4.3	−18.1 ± 4.2	
Apex	−22.4 ± 6.1		

**Table 4:** Late enhancement myocardial segments (N, % of LE) by CMR/CCT (57 patients).

	Basal	Mid	Apical
Anterior	14 (24.5%)	15 (26.3%)	15 (26.3%)
Anteroseptal	9 (15.7%)	10 (17.5%)	9 (15.7%)
Inferoseptal	11 (19.2%)	16 (28.0%)	
Inferior	20 (35.0%)	23 (40.3%)	19 (33.3%)
Inferolateral	20 (35.0%)	24 (42.1%)	22 (38.5%)
Anterolateral	22 (38.5%)	27 (47.3%)	
Apex	9 (15.7%)		

CMR: cardiac magnetic resonance. CCT: cardiac computed tomography.

**Table 5:** Cut-off values for LV regional strain to predict LGE/LIE on CMR/CCT.

	Basal	Mid	Apical
Anterior	>−12.5%	>−12.9%	>−14.5%
Anteroseptal	>−12.9%	>−13.5%	>−18.5%
Inferoseptal	>−13.5%	>−12.5%	
Inferior	>−10.5%	>−12.5%	>−16.5%
Inferolateral	>−11.5%	>−14.5%	>−14.2%
Anterolateral	>−12.5%	>−14.5%	
Apex	>−18.5%		

The cut-off values were established using the Receiver Operating Characteristics curves, using the Youden index (Supplementary File). LV: left ventricle. LGE: late gadolinium enhancement. LIE: late iodine enhancement. CMR: cardiac magnetic resonance. CCT: cardiac computed tomography.

**Table 6:** Univariate logistic regression of regional left ventricular strain to predict the likelihood of LGE/LIE.

LV Regional Strain/Each Segment)	OR	95%CI	*p*-Value
Strain segment 1 > −12.5%	3	0.7–11.9	0.11
Strain segment 2 > −12.9%	2.3	0.3–14.5	0.3
Strain segment 3 > −13.5%	0.6	0.07–6.1	0.7
Strain segment 4 > −10.5%	1.4	0.2–7.0	0.6
Strain segment 5 > −11.5%	0.7	0.1–4.0	0.7
Strain segment 6 > −12.5%	1.2	0.2–6.0	0.8
Strain segment 7 > −12.9%	2.3	0.4–12.1	0.2
Strain segment 8 > −13.5%	1.7	0.2–10.0	0.5
Strain segment 9 > −12.5%	2.4	0.5–10.4	0.2
Strain segment 10 > −12.5%	1.1	0.2–5.5	0.8
Strain segment 11 > −14.5%	1.8	0.5–6.1	0.3
Strain segment 12 > −14.5%	7	1.3–36.1	0.02
Strain segment 13 > −14.5%	3.7	0.8–15.3	0.07
Strain segment 14 > −18.5%	2.4	0.5–11.8	0.2
Strain segment 15 > −16.5%	1.9	1.0–14.4	0.04
Strain segment 16 > −14.2%	1.1	0.3–4.3	0.8
Strain segment 17 < −18.5%	5.1	1.1–23.1	0.03

LV: left ventricle. LGE: late gadolinium enhancement. LIE: late iodine enhancement.

## Data Availability

Data supporting the findings of this study are not available due to privacy restrictions.

## Supplementary Materials

The following supporting information can be downloaded at: https://www.mdpi.com/article/10.3390/jcm13237436/s1, Figure S1: ROC curves illustrating the strain cut-off values used to predict the presence of Late Enhancement (LE) in Cardiac Magnetic Resonance imaging (CMR). These thresholds were derived from our analysis to identify significant correlations between strain values and myocardial fibrosis in patients with Arrhythmogenic Cardiomyopathy (ACM).
