# Supplementary figures and images for: Strain Analysis for Early Detection of Fibrosis in Arrhythmogenic Cardiomyopathy: Insights from a Preliminary Study
Source: J Clin Med. 2024 Dec 6;13(23):7436. doi: 10.3390/jcm13237436 (PMC11642217; doi:10.3390/jcm13237436)

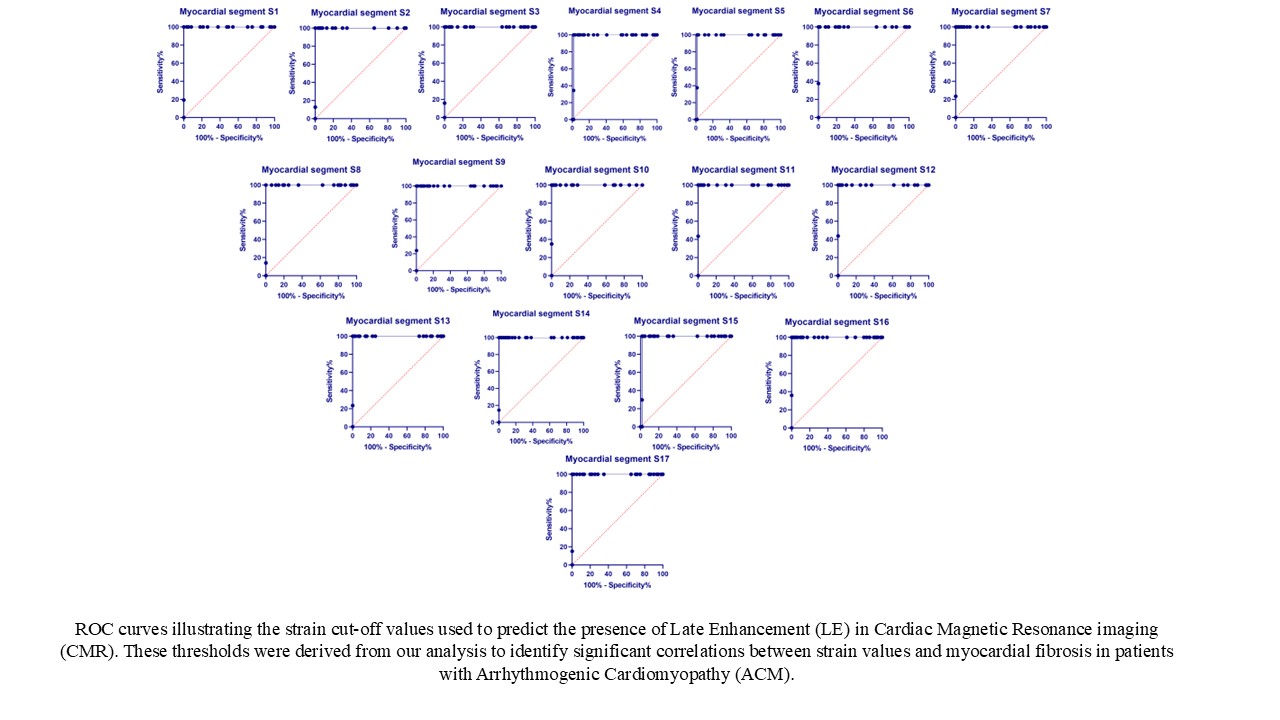

Supplement: Supplementary file 1 [file jcm-13-07436-s001.zip › jcm-3337588-supplementary.jpg]
